# Condensing Raman spectrum for single-cell phenotype analysis

**DOI:** 10.1186/1471-2105-16-S18-S15

**Published:** 2015-12-09

**Authors:** Shiwei Sun, Xuetao Wang, Xin Gao, Lihui Ren, Xiaoquan Su, Dongbo Bu, Kang Ning

**Affiliations:** 1 Key Lab of Intelligent Information Processing, Institute of Computing Technology of the Chinese Academy of Sciences, 100190 Beijing, China; 2 CAS Key Laboratory of Biofuels and Shandong Key Laboratory of Energy Genetics, Bioinformatics Group of Single-Cell Center, Qingdao Institute of Bioenergy and Bioprocess Technology, Chinese Academy of Sciences, Qingdao, 266101 Shandong, P. R. China; 3 CUDA Research Centre of Qingdao, Qingdao, 266101 Shandong, China; 4 Computational Bioscience Research Center, King Abdullah University of Science and Technology, 23955-6900, Thuwal, Kingdom of Saudi Arabia; 5 College of Life Science and Technology, Huazhong University of Science and Technology, Wuhan, 430074 Hubei, China

**Keywords:** Raman Spectrum, Linear Discriminant Analysis (LDA), K Nearest Neighbor(k-NN), Discretization

## Abstract

**Background:**

In recent years, high throughput and non-invasive Raman spectrometry technique has matured as an effective approach to identification of individual cells by species, even in complex, mixed populations. Raman profiling is an appealing optical microscopic method to achieve this. To fully utilize Raman proling for single-cell analysis, an extensive understanding of Raman spectra is necessary to answer questions such as which filtering methodologies are effective for pre-processing of Raman spectra, what strains can be distinguished by Raman spectra, and what features serve best as Raman-based biomarkers for single-cells, etc.

**Results:**

In this work, we have proposed an approach called rDisc to discretize the original Raman spectrum into only a few (usually less than 20) representative peaks (Raman shifts). The approach has advantages in removing noises, and condensing the original spectrum. In particular, effective signal processing procedures were designed to eliminate noise, utilising wavelet transform denoising, baseline correction, and signal normalization. In the discretizing process, representative peaks were selected to signicantly decrease the Raman data size. More importantly, the selected peaks are chosen as suitable to serve as key biological markers to differentiate species and other cellular features. Additionally, the classication performance of discretized spectra was found to be comparable to full spectrum having more than 1000 Raman shifts. Overall, the discretized spectrum needs about 5storage space of a full spectrum and the processing speed is considerably faster. This makes rDisc clearly superior to other methods for single-cell classication.

## Introduction

Bacteria, plants, animals, and all other organisms on the planet are derived from or composed of single cells. Genetically identical parent cells can produce cells with different functions due to the intrinsic variation among the individual offspring cells in gene structure, gene expression, and gene regulation[1,2]. By monitoring microbial single cells *in vitro *along the time course and under varying conditions, we could effectively analyze how a population adapts to ever-changing conditions [3], such as those regarding nutrient supply or stress exposure[4]. Microbiologists are especially interested in techniques centered on single cells because they serve as the basic unit of functional microorganisms, yet most microorganisms (*>*99%) have not yet been cultivated in the laboratory[5]. There is increasing evidence that these uncultured microorganisms play crucial roles in ecosystems and have a pro-found impact on global warming (through carbon/nitrogen cycles)[6], food security (through maintaining soil heathland-promoting plant growth)[7], and environmental bioremediation[8].

In studying microorganisms, therefore, there is great promise of gaining substantial insight into fundamental physiological processes in microorganisms and of accelerating the development of superior strains for industrial biotechnology.

Single-cell technologies, such as the classical fluorescence-activated cell sorting (FACS) analysis [9] and the more recently developed Raman spectra profiling, can detect population diversity by observing distinct phenotypic parameters. Raman spectroscopy is an especially powerful analytical technique and has already been used in several studies on single cells [10]. Raman spectroscopy is based on inelastic scattering of photons following their interaction with vibrating molecules of the sample. During this interaction, photons transfer (Stokes)/receive (Anti-Stokes) energy to/from molecules as vibrational energy. Thus the energy change of the scattered photons corresponds to the vibrational energy levels of the sample molecules. A single-cell Raman spectrum usually contains more than 1,000 Raman shifts, which provide rich information of the cell (e.g., nucleic acids, protein, carbohydrates and lipids), reflecting cellular genotypes, phenotypes, and physiological states [11]. Therefore, a Raman spectrum could serve as a molecular ?fingerprint? of a single cell, enabling the distinction of various cells, including those from bacteria and animals, without prior knowledge of the cells (details about Raman spectroscopy can be found at [12]).

**Figure 1:**
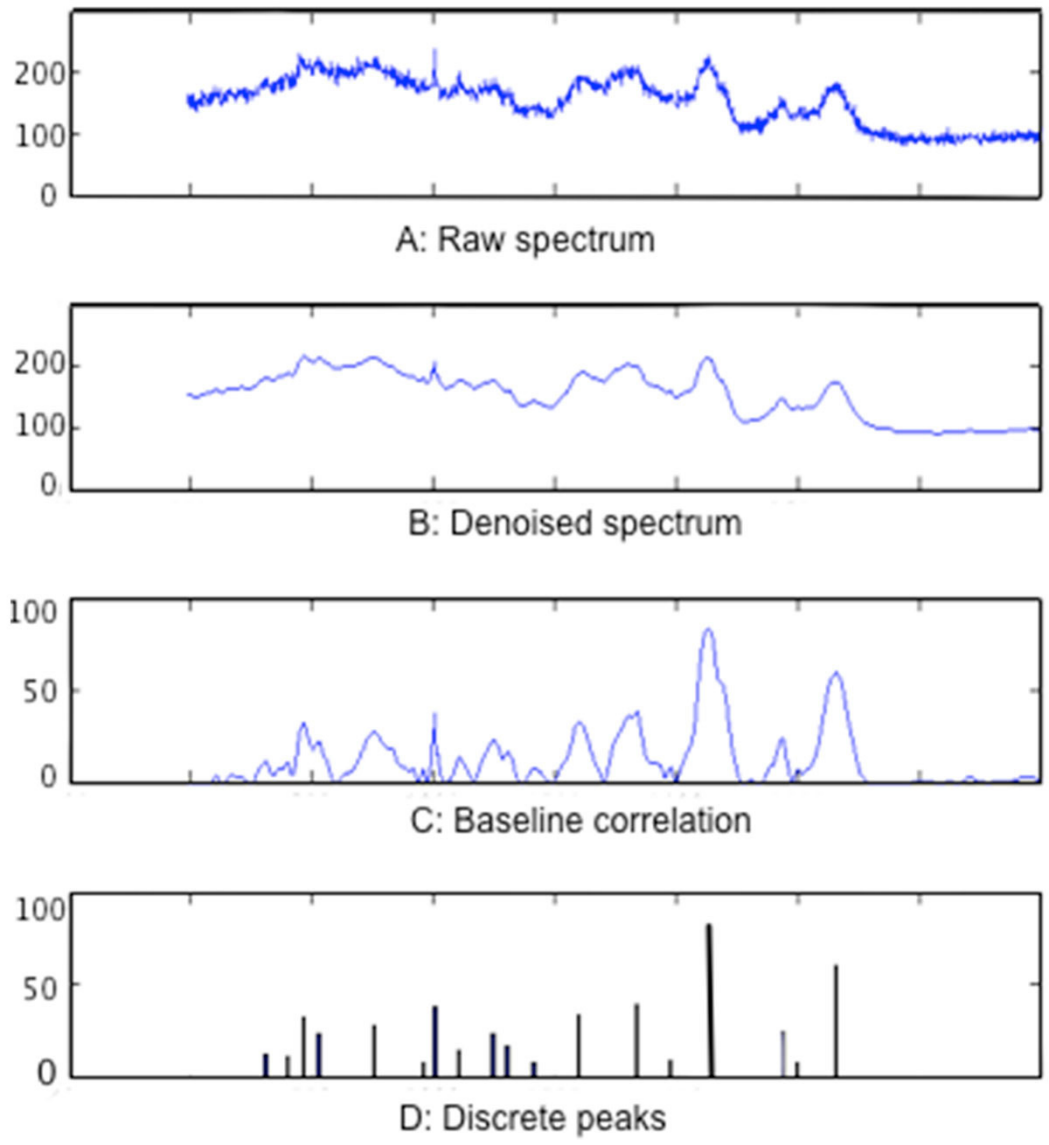
**An illustration of single-cell Raman quality control and discretization results**. (A) represents the raw Raman spectrum, (B) represents the Raman signal after wavelet denoising, (C) is the result of baseline correction, and (D) is the Raman spectrum discretization.

Raman spectra are continuous data, which are not easily used and interpreted[13] and which prevent many induction algorithms that require discrete data. In contrast, discrete values have many advantages (e.g., rules with discrete values are normally shorter and more understandable, and discretization can lead to improved predictive accuracy). Furthermore, many induction algorithms found in literature require discrete values. All of these points prompt us to establish a data processing flow to discretize continuous Raman spectrum to discrete spectrum, named rDisc (Raman spectrum discretization), including quality control and discretization, as shown in Figure 1. We also evaluated classification performance based on the discretization spectrum for single cells from different strains using the two most popular classification algorithms: linear discriminant analysis (LDA) and k-nearest neighbor (k-NN). Test results from these two classification algorithms showed that classification performance based on discretized spectrum (with ~20 representative peaks) could achieve accuracy comparable to full spectrum with more than 1,000 Raman shifts. More importantly, the storage space needed by a discretized spectrum is only about 5 percent of that needed by a full spectrum, and the processing speed is much faster.

## Materials and methods

### Single-cell Raman spectrum datasets

For this study, we selected 3,678 Raman spectra from five microorganisms for quality control and clustering analysis, which are available from QSpecDB database at http://www.computationalbioenergy.org/QSpec/homepage.htm These five single-cell microbes are *Corynebacterium glutamicum, Chlamydomonas reinhardtii, Chlorella pyrenoidosa, Thermoanaerobacter sp. X514, and E.coli DH5α*. These microorganisms differ in biological background and physiological status, making the comparison between them unbiased and representative (as shown in Table 1). For Raman spectra capturing, we we employed laser with a wavelength 532nm. Based on the experimental requirements of different cells, we used different acquisition times (0.01, 0.05, 0.1, 0.2, 0.5, 1, 5, and 30s) at the single-cell level and made sure the S/N ratio fell within the appropriate range[14].

**Table 1:** Single-cells Raman datasets used in this work.

ID	Name	Number of cells
1	*Corynebacterium glutamicum*	42
2	*Chlamydomonas reinhardtii*	237
3	*Chlorella pyrenoidosa*	1178
4	*Thermoanaerobacter sp. X514*	197
5	*E.coli DH*	143

Laser with a wavelength 532 nm has been used for Raman spectra capturing. Species representing a wide range of tree-of-life microorganisms have been chosen; among these species, some have a larger number of single-cell Raman spectra for more in-depth analysis.

### Analysis methods

The rDisc includes two main parts: Raman quality-control procedure and Raman discretization via representative peaks. They are described, respectively, in the following sections.

#### Quality control

The signals of Raman spectroscopy are weaker than others used during the signal acquisition, though surface-enhancing technology has been adopted to strengthen the energy of Raman signals[15,16]. The Raman signals inevitably mix with several other components with energy a few orders higher, such as intrinsic fluorescence signals, and the random instrument noises, etc. Therefore, quality-control methods should be adopted for Raman spectrum preprocess.

In Figure 1, a three-step approach for quality control is defined to get a relatively high-quality spectrum: wavelet transform denoising, baseline correction, and normalization.

*Discrete wavelet transformation denoising *Due to the change of voltage, current and other instrument parameters, electromagnetic noise (such as Gaussian noise), and impulse noise are inevitably brought into the Raman spectrum, lowering the signal-noise ratio of Raman spectra. In this study we have used the discrete wavelet denoising method, which is suitable for complicated Raman spectra [17,18].

**Figure 2:**
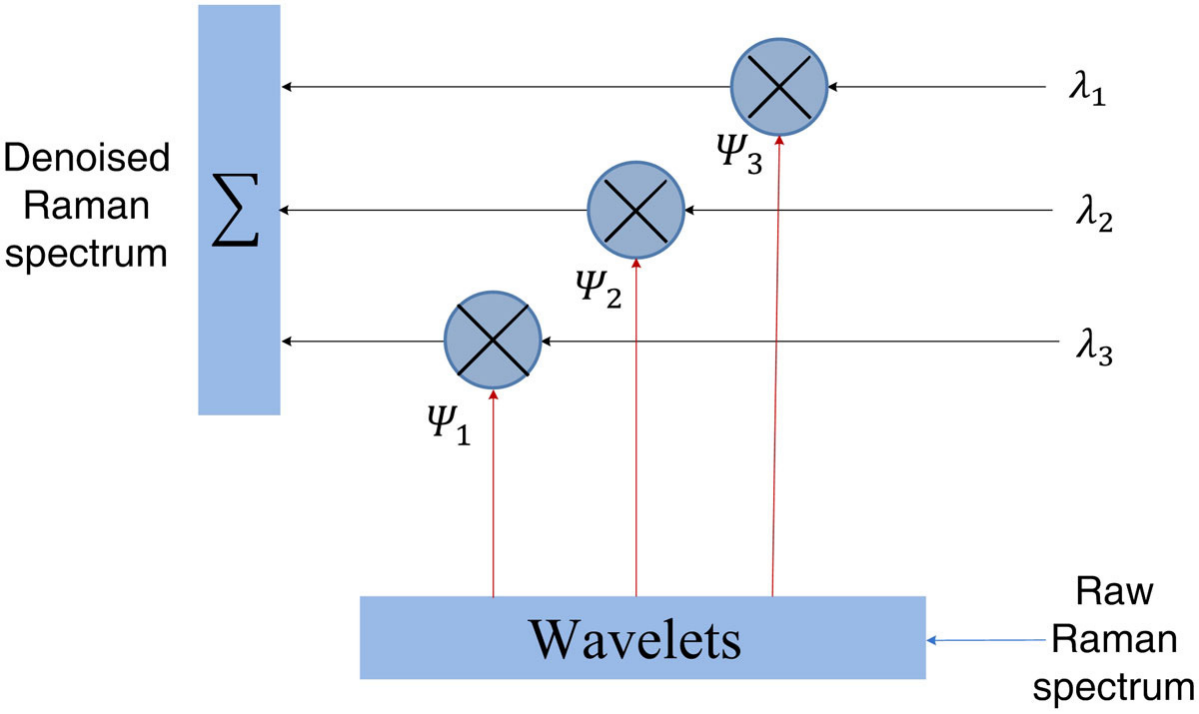
**Raman spectrum discrete wavelet denoising**.

As with the Fourier transform method, discrete wavelet transform offers effective time-frequency analysis[19,20]. It can analyze the signals at the domains of frequency and time simultaneously. Using this method, Raman signals are first deconstructed by discrete wavelet transform to obtain the differing frequency-domain information, and then each domain coefficient is adjusted to reconstruct the Raman signals, primarily by decreasing the coefficient in the noise level. Given the fact that noise generally spreads in the higher deconstruction level, lowering the coefficient in this level can effectively reduce noise. Based on experience we have selected three order discrete wavelet transform for noise reduction in this study (Figure 2). As shown in Figure 1(B), the denoised Raman signal by discrete wavelet transform is smoother and still retains most significant features of Raman signals.

*Baseline correction *The Raman spectrum baseline comes from the intrinsic back-ground signals [21], which always interfere with and even submerge the weak Raman signal. The baseline is generally much more intense than Raman signals and usually appears as low-frequent smooth curve. To mitigate the negative effect, it is necessary to correct the baseline Raman shift[22,23]. To date, many baseline correction methods have been proposed, including (a) one-order or two-order derivation, (b) single or modified multi-polynomial fitting, and (c) wavelet transformation, widely used in the field of signal processing [24,25]. However, for Raman spectrum with complicated background noise, these methods might not work well, possibly requiring certain user interference or taking a long time. In this study we have developed an automatic piecewise fitting method for Raman baseline correction.

First, an automatic algorithm detects the trough positions $\left(w_{1},\;w_{2},\;\cdots \;,\;w_{n}\right)$ in a Raman spectrum; then each adjacent wave trough is fitted by a low-order fitting curves; and finally, all these fitted curves are connected to form the whole baseline. The result of correcting a Raman spectrum baseline is shown in Figure 1(C). It can additionally largely reduce the computational time entailed by other methods.

*Normalization *Raman signal normalization effectively removes the effects of different instrument parameter settings, such as voltage and signal offset. This normalization could render signals of different scale levels comparable while leaving the shape of Raman signals unaffected. Of the many normalization methods suitable for the Raman spectrum process, we have utilized the linear function method (eq.1) to normalize the Raman spectrum, in which *min*(*x*) and *max*(*x*) represent, respectively, the minimum and maximum intensity values in a Raman spectrum, in which *Y *represents one Raman spectrum and the *yi*represents one Raman shift in the *Y*.

(1) $$y_{i}=\frac{y_{i}-min\left(Y\right)}{max\left(Y\right)-min\left(Y\right)}$$

### Raman spectrum discretization

Certain applications, such as Raman spectrum classification and searching for a large amount of spectra, are inefficient in light of the more than 1,000 spatial resolutions (Raman shifts) of Raman spectra (Figure 1(c)). Methods such as principal component analysis (PCA) and linear discriminant analysis (LDA) effectively reduce dimensionality of Raman spectra [26], However, while data dimensions in PCA and LDA analyses are linear combinations of all dimensions of original data, no individual dimension has special meaning. For single-cell Raman spectra, it was worth noting that some Raman shifts would have special biological meaning and might correspond to one or a set of compound structure(s) in the cell. Therefore, selecting representative peaks from all Raman shifts would be more suitable for dimension reduction of Raman spectrum (Figure 1(D)).

**Figure 3:**
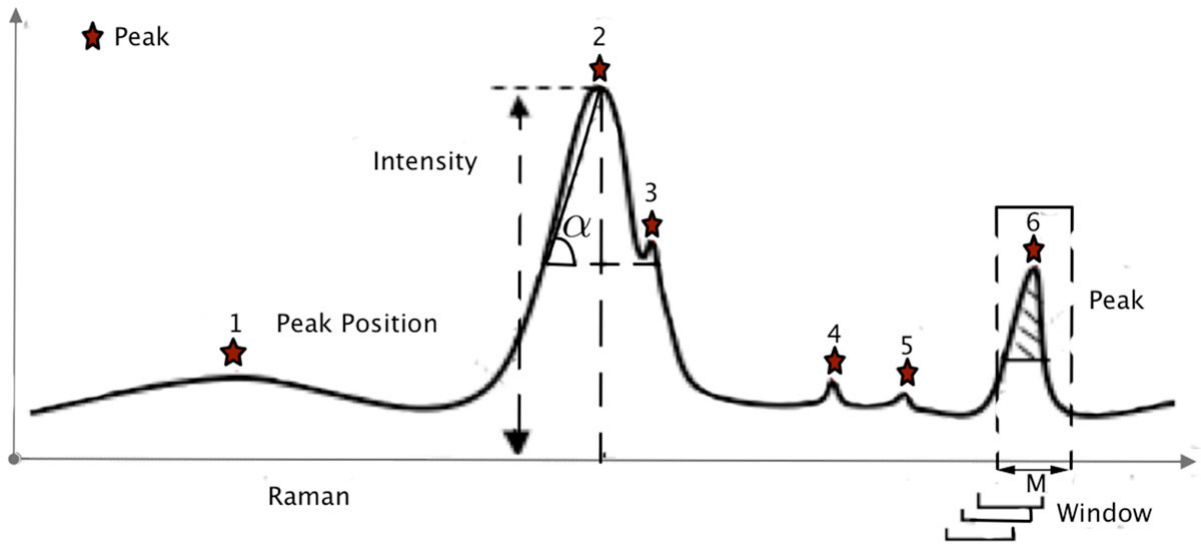
**Principles for Raman spectrum discretization**. Stars represent peaks, and dashed boxes represent windows.

We have designed an automatic peak-detection algorithm to recognize the positions of Raman peaks positions as well as to remove peaks that may originate from autofluorescence background signals, dark noise, and instrument response with negative effect to further application, such as classification analysis in Figure 3.

To accomplish this, we first defined a sliding window with a width of *M *Raman shifts, traversing through the whole Raman spectrum, and when the intensity in the center of the widow is larger than other points in this window, this center was considered as one candidate peak. In this way we can remove those small peaks such as peak "3" in Figure 3. Based on experience, we have set the value of *M *= 20 in this study. Secondly, we have calculated tan(*θ*) (in Figure 3 ) for each peak in step 1 to represent the peak sharpness, and when tan(*θ*) was less than threshold *α*, the peak (as peak "1" in Figure 3) too flat compared to other Raman peaks, would be moved.

Thirdly, the average noise level in a Raman spectrum was also calculated. For example, suppose that a Raman spectrum has *N *peaks. (1) We defined a constant *M *that could divide the Raman spectrum into consecutive *N *− *M *+ 1 widows. (2) In each window we calculate the window-wise average intensity and variant. (3) We selected a window with minimum average intensity and below-threshold variant. We have defined the average intensity of this window as the "noise" level. Thus there is a peak in a window when the window satisfied the following conditions:

(2) $$p_{i}>max\left(p_{i-M},\;\cdots \;,\;p_{i+1},\;\cdots \;,\;p_{i+M}\right)|k\left(i\right)>\lambda |p_{i}>\;\text{noise}$$

(3) $$k=\frac{\left|f^{\prime \prime }\left(x\right)\right|}{{\left(1+{f{}^{\prime }}^{2}\left(x\right)\right)}^{3/2}}$$

The peak detection results in a discretization method (Figure 3) that can reduce the Raman spectrum size by more than 100 times. Thus the use of discretized spectrum, with its small amount of data, can significantly reduce time spent searching an unknown sample. Additionally, for selected peaks, or features, from single-cell Raman spectra, three pieces of base information could be used: peak position, peak intensity (height), or peak area information. The Raman spectrum after discretization process was referred to as 'discrete Raman spectrum', and we have also defined the rDisc format for these discrete spectrum. In rDisc format, each row constitutes a representative peak, with its shift position, intensity, and curate value (represent the peak's sharpness) provided. Single-cell Raman spectra in this format will be used in following analyses.

### Similarity measurement between discrete spectra

The classification analysis was designed to evaluate whether the discretized spectrum could effectively represent the full spectrum. Accordingly, we defined some rules, by the discrete Raman spectra, to calculate the similarity of two Raman spectra, including peak position matching, peak intensity correlation, combination of peak position matching, and intensity correlation.

*Peak position matching ratio *In this type of ratio, the similarity of two Raman spectra is calculated based on their peak position information. In eq.(4), *p_i _*and *p_j _*represent the sets of peak positions (Raman shift) of two Raman spectra. The numerator calculates the number of common peaks between *p_i _*and *p_j_*, while the denominator calculates the number of the union set of *pi *and *pj *. In addition, based on experience, two peaks are considered as 'matched' when their positions differ by, at most, 10 Raman shifts(*cm^−1^*).

(4) $$s_{1}=\frac{p_{i}\cap p_{j}}{p_{i}\cup p_{j}}$$

*Peak intensity similarity evaluation *In eq.5, peak intensity is utilized in addition to peak-position information to increase the reliability of similarity evaluation, in which the Pearson correlation was selected as the method for evaluating intensity of the peaks. As the different Raman spectra have different numbers of candidate peaks, the common peak intensity (*v_i_, v_j _*in eq.5) in two Raman spectra will be used to calculate the Pearson correlation value. Again, two peaks are considered 'matched' when their positions differ by, at most, 10 Raman shifts(*cm^−1^*). For those Raman spectra with no common peaks, the Pearson correlation value is simply defined as 0, denoting no correlation.

(5) $$s_{2}=\frac{\sum \left(v_{i}-{\bar{v}}_{i}\right)\;\left(v_{j}-{\bar{v}}_{j}\right)}{\sqrt{\sum {\left(v_{i}-{\bar{v}}_{i}\right)}^{2}\;{\left(v_{j}-{\bar{v}}_{j}\right)}^{2}}}$$

*Combined approach *To increase the accuracy of similarity eq.4 and eq.5 are combined to form eq.6. We have combined the square root of *s*_1 _with *s*_2 _based on our experience in data manipulation. As a result, eq.6 can be used as a similarity function to recognize the unknown sample's category, such as k-NN.

(6) $$s=\sqrt{s_{1}*s_{2}}$$

### Availability of Raman spectrum data analysis method

We have integrated the above quality-control and discretized methods into a data analysis package named rDisc, available at: http://www.computationalbioenergy.org/imod.html All single-cell Raman spectra in raw data format as well as in rDisc format (representing discrete spectrum) have been stored or linked on this website.

## Results and discussions

### Evaluation of classification performance of discretized spectra

We performed experiments to evaluate the single-cell classification performance of a Raman discretized spectrum, compared to those based on full spectra. All Raman spectra were processed by the whole quality-control procedure. The five species from the QSpec Raman database were randomly divided into training group or test group by the separation ratio of 60%:40%. The separation and analysis has been repeated 10 times.

We have selected several classical methods for comparison, including k-NN (k-nearest neighbour) based on full-spectra (using Pearson correlation for similarity measurement), LDA based on full-spectra, k-NN based on discretized Raman spectra, and LDA based on discretized Raman spectra. In the last of these methods, we used discretized spectrum as the LDA input data. To ensure the consistent data dimension for LDA, all unique peaks in training spectra were detected and saved in an array, and only positions in this array would be considered for test Raman spectra. The comparison results of different classification methods were shown in Table 2, from which we could observe that the method based on discretized spectra showed better classification performance by k-NN(0.95) and LDA(0.95), indicating that discretized spectra can represent the information of full Raman spectra for classification.

**Table 2:** Comparison of different clustering methods.

	Full Spectrum	Discretized Spectrum		
**Methods**	**k-NN**	**LDA**	**k-NN**	**LDA**

	0.95	0.68	0.95	0.95

### Efficiency evaluation

Processing speed for single-cell Raman spectrum classification, with the rapidly increasing number of Raman spectra, could be a critical concern. rDisc based on discretized spectra has naturally enabled the efficient processing of single-cell Raman spectra as data size was reduced greatly. Here we have evaluated the execution efficiency of k-NN based on discretized spectra. We conducted all of these experiments on the computer with Intel Xeon E3-1225 CPU (4 cores in total, 3.2GHz), 4GB DDR3 ECC RAM, and 500GB hard drive. We have discovered that the classification time for each Raman spectrum in the dataset with 300 randomly selected Raman spectra from QSpec-DB was 0.009s for k-NN based on discretized spectra and approximately 0.051s for k-NN based on full spectra. Such a speed-up, of more than five times, would be especially useful in circumstances involved a large volume of single-cell Raman spectra.

## Discussion

There are several advantages to the discrete spectrum for single-cell Raman spectrum analysis, including speed, accuracy, and biomarker identification. It would therefore be very valuable across a variety of applications. First, its application in single-cell sorting could enable near real-time database comparison and search?due to its fast speed, culled from peak discretization, and high accuracy regarding Raman spectrum comparison. Thus, it would be especially suitable for single-cell profiling and large-scale sorting [27].

Second, it could be used for microbial community analysis based on single cells, in which single cells from hundreds or even thousands of unknown different strains need to be analyzed separately. Based on a large collection of single-cell Raman spectra, as well as its high accuracy for single-cell classification [28], rDisc could be used for such applications.

Third, discrete spectrum could be easier and more understandable to use for diagnosis based on single cells, as it could accurately identify biomarkers out of millions of single-cell Raman spectra. This would be especially useful in clinical application such as the diagnosis of early-stage cancer[29].

In applied settings, a method should classify closely related species based on Raman spectra data. However, the number of species with single-cell Raman spectra is currently small, meaning we do not have enough species to test the extent to which our method can classify closely related species. Given current results, we could say, already, that our method could accurately differentiate single cells based on their Raman spectra. We hope to soon obtain enough data to test it with the advancement of single-cell Raman spectrometry techniques.

## Conclusions

Single-cell phenotype analysis, such as Raman spectrum analysis, is growing in popularity correspondent to increasing concerns and focus on single-cell analysis and applications. However, current single-cell Raman spectrum analysis is still limited by the lack of data model, data quality control, and high-performance data analysis methods.

In this work, we have proposed a single-cell Raman spectrum analysis method, named rDisc, to transfer a native Raman spectrum to a discrete spectrum, thus enabling the reasonably efficient and accurate analysis of a huge number of single cells. The results have shown discrete spectrum could improve the quality of Raman spectra and the classification accuracy. Second, single-cell Raman spectrum discretization would improve the speed of spectrum comparison. Third, representative peaks could be identified to differentiate certain species from others based on discrete spectra by rDisc, which could serve as biomarkers for that species. Having enabled all of these advanced features for single-cell Raman spectrum analysis, the rDisc method represents the latest advancement for single-cell phenotype analysis in the era of "big-data". Considering the ever-increasing number of single-cell Raman spectrum data, rDisc could be improved on two ends?speed and depth of data-mining. The speed of the current classification method could be further improved by utilizing the latest development of high-performance computation techniques such as GPU computation, as well as refined spectrum filtration method for their similarity assessment.

Given the huge number of different types of single-cells phenotype, deep datamining, including self-organizing map (SOM) and Bayesian network, could be used to analyze the relationships/redundancies between peaks, as well as refined clustering of single cells.

## Competing interests

The authors declare that they have no competing interests.

## Authors' contributions

SS and XW performed the statistical analysis and drafted the manuscript. LR and XS carried out the experimental analysis and assisted with the drafting of the manuscript. XG assisted with the interpretation of the data and critically revised the manuscript. KN and DB conceived of the scientific studies and participated in their design and coordination. KN conceived of the statistical methodology and helped to critically revise the manuscript. All authors read and approved the final manuscript.

## Declarations

Publication charges for this article have been funded by National Science Foundation of China grant No.31270834.

This article has been published as part of *BMC Bioinformatics *Volume 16 Supplement 18, 2015: Joint 26th Genome Informatics Workshop and 14th International Conference on Bioinformatics: Bioinformatics. The full contents of the supplement are available online at http://www.biomedcentral.com/bmcbioinformatics/supplements/16/S18.
